# Ionic Liquid-Assisted Hydrothermal Synthesis of a Biocompatible Filler for Photo-Curable Dental Composite: From Theory to Experiment

**DOI:** 10.3390/ma12142339

**Published:** 2019-07-23

**Authors:** Kh. Moradi, A.A. Sabbagh Alvani, D. Poelman

**Affiliations:** 1 Color & Polymer Research Center, Amirkabir University of Technology (Tehran Polytechnic), P.O. Box 15875-4413, Tehran, Iran; 2 Department of Polymer Engineering and Color Technology, Amirkabir University of Technology (Tehran Polytechnic), P.O. Box 15875-4413, Tehran, Iran; 3 Advanced Materials Group, Iranian Color Society (ICS), P.O. Box 1591637144, Tehran, Iran; 4 Lumilab, Department of Solid State Sciences, Ghent University, B-9000 Ghent, Belgium

**Keywords:** Sr-doped hydroxyapatite, ionic liquid, hydrothermal, biocompatibility, DFT calculations

## Abstract

Nanostructured hydroxyapatite (HA) is a new class of biocompatible fillers which has been recently utilized in bio hybrid materials by virtue of its excellent tissue bioactivity and biocompatibility. However, the need for higher thermal stability, solubility, surface bioactivity, radiopacity, and remineralization ability suggests a divalent cation substitution of HA for use in light curable dental restorative composites. In this work, structural and optical properties of Sr-doped hydroxyapatite were studied using first-principle calculations based on density functional theory (DFT). Next, Sr-doped hydroxyapatite (HA) was prepared via a new ionic liquid-assisted hydrothermal (ILH) route. Samples were characterized using X-ray diffraction (XRD), scanning electron microscopy (SEM)/energy dispersive spectroscopy (EDS), Fourier transform infrared spectroscopy (FTIR), transmission electron microscopy (TEM), dynamic light scattering (DLS), Brunauer–Emmett–Teller (BET) surface area analysis, and cell viability. The obtained experimental data showed that the nucleation and crystal growth process controlled by [BMIM]Br molecules results in uniform products with small and regular particles and high specific surface areas. Finally, cytotoxicity tests showed that the as-prepared Sr-doped HA nanoparticles have good biocompatibility (≥91%), confirming their potential for use in photo-curable dental restorative composites.

## 1. Introduction 

Hydroxyapatite (HA, Ca_10_(PO_4_)_6_(OH)_2_) is one of the most promising bioactive materials for the production of artificial bone grafts and teeth joints with good osteoconductive and biocompatible properties for biomedical applications [1,2,3]. In order to explore the effects of calcium substitution by metals in synthetic HA, various works have already been published [4,5,6,7]. The substitution of divalent cations, such as Sr for Ca, in the lattice structure of HA modifies its thermal stability, solubility, and textural properties, as well as its surface reactivity. Moreover, these substitutions can also significantly promote the biological response, radiopacity, and remineralization ability of pure HA for dental restorative composite applications [8,9,10,11,12]. Several in vitro studies demonstrated that collagen type I production and remineralization of caries lesions can be facilitated by the release of strontium from dental composites [13,14].

To synthesize hydroxyapatite nanostructures, several approaches have been reported, such as chemical precipitation [15], microwave assisted synthesis [16,17], mechano-chemical synthesis [18], micro-emulsion [19], and sol-gel [20]. Among these, the hydrothermal method has been considered as one of the most favorable methods to prepare HA nanostructures due to the possibility of achieving highly crystalline products, high efficiency, relatively simple and cost-effective synthesis, controllable morphology, and a Ca/P ratio close to stoichiometric HA [21,22]. Furthermore, due to the potential formation of nanostructures with tunable morphology, lower agglomeration, and higher surface area, organic surface modifiers have been used in the synthesis of nanoparticles [23,24]. Ionic liquids (ILs) have recently been widely employed in the preparation of inorganic materials with unique characteristics because of their desirable chemical and physical properties, such as high ionic conductivity, high polarity, and low toxicity [25,26,27]. Ionic liquids can function as either templating agents or for capping/morphology-controlling. Thereby, they have the ability to control the nucleation and growth of the nanostructures, thus inhibiting the aggregation of the resultant nanoparticles through electrostatic interaction (due to their intrinsic charge), steric hindrance (by alkyl substituent), and electro-steric stabilization (due to the amphiphilic structure of ILs) [28,29]. In recent years, modified hydro- and solvo-thermal synthesis using ILs were applied to synthesize various inorganic materials [30,31,32,33,34]. The results have revealed that the presence of an ionic liquid as a co-solvent and capping agent allows preparation of nanoparticles with desired physical and structural properties. To the best of our knowledge, hardly any studies were devoted to IL-assisted hydrothermal preparation of Sr-doped HA nanostructures. In this work, Sr-doped HA nanoparticles were successfully synthesized via an IL-assisted hydrothermal method and the phase purity, microstructure, and biocompatibility of the as-prepared products were comprehensively investigated. Moreover, for comparison, Sr-doped HA was also synthesized via the conventional hydrothermal (CH) route (without IL-based capping agents). 

## 2. Experimental Procedure

### 2.1. Materials Synthesis 

Strontium nitrate (Sr(NO_3_)_2_), Calcium nitrate (Ca(NO_3_)_2_·4H_2_O), diammonium hydrogen phosphate ((NH_4_)_2_HPO_4_), ionic liquid [BMIM]Br, and ammonium hydroxide (NH_4_OH) were purchased from Sigma-Aldrich and used without further purification. The HA nanoparticles were synthesized by an IL-assisted hydrothermal method. Ca(NO_3_)_2_·4H_2_O, with an appropriate amount of strontium nitrate (Sr concentration: 5 mol%) and (NH_4_)_2_HPO_4_ were separately mixed with double distilled water with a Ca/P ratio of 1.67. In the calcium containing solution, 1.5 mmol [BMIM]Br ionic liquid was added as a capping agent. A NH_4_OH solution was added gradually to the phosphate solution to adjust the pH level to 8. After continuous stirring for 10 min, the obtained solution was transferred to a Teflon container autoclave, placed in the furnace, and heated for 12 h at 180 °C. Finally, the obtained precipitates were washed and dried at 80 °C and further calcined at 600 °C for 2 h in air.

### 2.2. Models and Computational Details

All calculations were performed based on first-principles DFT using the Cambridge Serial Total Energy Package (CASTEP), as implemented in Materials Studio [35]. The exchange-correlation interactions are treated at the generalized gradient approximation (GGA) level, employing the Perdew–Burke–Ernzerhof (PBE) functional [36]. The Ca *3s^2^ 3p^6^ 4s^2^* electrons, P *5d^2^ 6s^2^* electrons, O *2s^2^ 2p^4^* electrons, H *1s^1^* electrons, and Sr *4s^2^4p^6^5s^2^* electrons were treated as valence electrons. In this study, a unit cell modification suggested by Mostafa et al. [37] was employed for HA. Brillouin-zone integrations were performed using Monkhorst and Pack *k*-point meshes (3 × 4 × 4) to obtain the accurate density of the electronic states [38,39,40,41]. The kinetic energy cutoff for wave function expansion was 340 eV and the self-consistent field (SCF) tolerance was set at 10^−6^ eV/atom. The convergence criterion of total force acting on each atom was considered as less than 0.01 eV/Å. The maximum displacement and stress were 10^−4^ nm and 0.02 GPa, respectively. The standard deviations were all less than 1%, which can confirm the reliability of our calculations for both crystal structures.

### 2.3. Materials Characterization

All samples were characterized using Fourier transform infrared spectroscopy (FTIR, Perkin Elmer Spectrometer, Waltham, MA, USA), X-ray diffraction (XRD, Inel, EQuniox 3000, Stratham, NH, USA, with Cu Kα radiation, λ = 0.154 nm), scanning electron microscopy (SEM/EDS, Seron Technologies AIS2100, Uiwang-si, Korea), transmission electron microscopy (TEM, Philips EM 208S, Beaverton, OR, USA), and N_2_ physi-sorption (Micromeritics ASAP 2010, Norcross, GA, USA). Moreover, for determining particle size distributions via dynamic light scattering (DLS), a Malvern Zetasizer Nano S (Malvern, UK) was used. Powders were dispersed in ethanol and sonicated for 20 min prior to the latter measurement. 

### 2.4. Cell Viability Measurements 

To evaluate the cytotoxicity of the as-synthesized sample, a breast cancer cell line (MCF-7) was used according to our previous work and the ISO 10993-5 protocol [42,43]. The MTT assay was used to determine the proliferation rate of the MCF-7 cells in the presence of extract powder. The powder suspension containing 0.1 g of powder was placed in 1 mL of culture medium and kept at 37 °C for predetermined time intervals. After 3, 7, and 14 days, the media were collected for use in a cellular test. The cells were cultured in RPMI with 10% (v/v) fetal bovine serum (FBS-serum, Gibco, Germany) 100 U·mL^−1^ penicillin and 100 μg·mL^−1^ streptomycin and then incubated at 37 °C in a humidified atmosphere in the presence of 5% CO_2_. The cells were cultured into a 96-well microtiter plate (Nunc, Denmark) at a density of 1 × 10^4^ cells/well. After 24 h, the culture medium in each well was removed and replaced by 90 µL extract powder plus 10 µL FBS. The medium was removed after 24 h and then 100 µL of 0.5 mg·mL^−1^ MTT (Sigma, USA) solution was added into each well. The cells were incubated for 4–5 h at 37 °C. The blue dark formazan crystals were observed and then dissolved by adding 100 μL of isopropanol (Sigma, USA) per well. The plate was incubated for 20 min before absorbance measurement.

## 3. Results and Discussion

**Figure 1:**
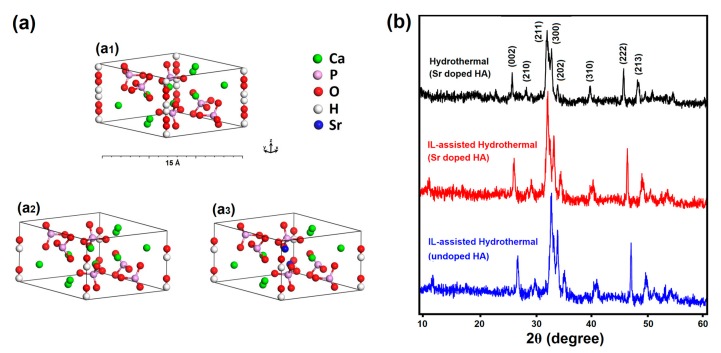
(**a**) Hexagonal primitive cell of HA and Sr-doped HA and (**b**) XRD patterns of the as-synthesized products.

The hexagonal primitive cell of doped and pure HA and the XRD patterns of the as-prepared Sr-doped HA samples synthesized via conventional and IL-assisted hydrothermal methods are shown in Figure 1. The results confirm the formation of the HA phase and all the diffraction lines correspond to the hexagonal structure of HA, according to the JCPDS card 09-0432. The hexagonal primitive cell of HA consists of tightly bonded PO_4_ tetrahedral units, two types of Ca atoms, and OH groups (44 atoms per unit cell with space group *P6_3_/m*) [44]. 

Due to the non-physical duplication of each OH group by the *m* plane, the symmetry was reduced to the *P6_3_* space group by removing two of the OH groups and placing the other two along the *c*-axis. This modification changes the space group to *P6_3_* and has been used before in many theoretical studies of HA [45,46]. The crystal structures of HA and Sr doped-HA with space group *P6_3_* are illustrated in Figure 1a_2_, a_3_. Furthermore, the lattice constants of the as-prepared Sr-doped and pure HA are calculated using XRD data and DFT calculations, listed in Table 1. The estimated lattice constants are in good agreement with earlier reports [9,10,47]. As expected, the larger atomic radius of Sr, compared to Ca, causes an increase in the lattice constants for the Sr doped-HA sample, compared to pure HA. A close inspection reveals that all the peaks ascribed to HA shift to lower 2θ values for the Sr-doped HA compound. According to Vegard’s law, if the lattice parameter increases in the presence of a dopant, then Sr ions enters the lattice of HA and cause lattice expansion. In other words, the large radius Sr ions (i.e., larger than that of Ca ions) can be the substitutes for Ca ions, resulting in the lattice distortion [48].

**Figure 2:**
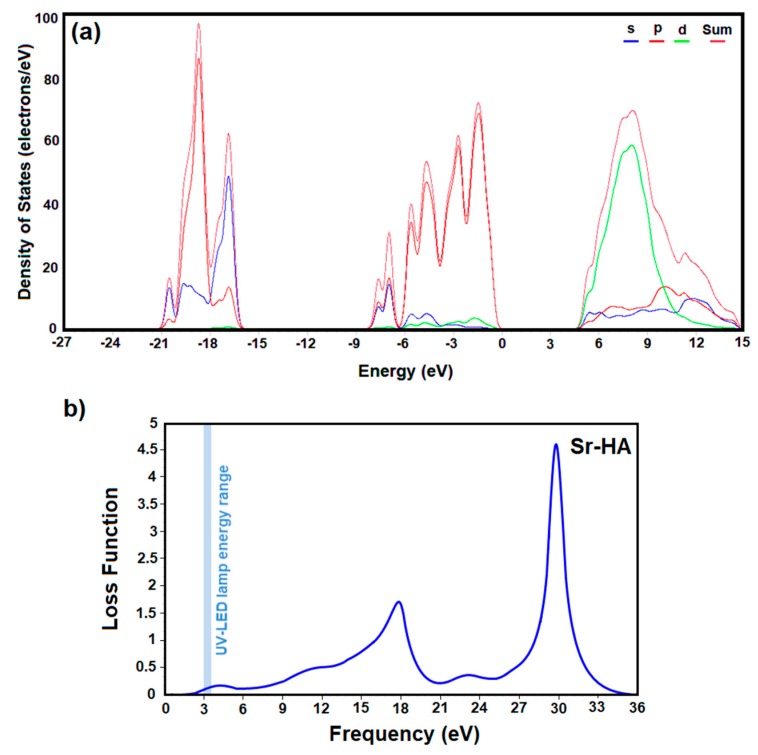
Electronic densities of states (**a**) and energy-loss spectra (**b**) of Sr-doped HA, obtained by DFT calculation.

To further study the underline physics of the elemental substitution, the density of states (DOS) for Sr-HA were calculated. As presented in Figure 2a, the top of the valence band ranging from −8 eV to 0 eV is almost exclusively contributed by oxygen *p* orbitals, while calcium *d* states are main constituents of conduction band. Due to substitution of Ca ions with Sr, the profile and peak intensity of the conduction band is slightly changed due to difference between the *d* state energy of calcium and strontium. 

The optical properties of the fillers used in light-curable dental composites is considered as a primary factor affecting curing depth, which is proportional to the physical properties and longevity of restorations. To investigate the optical characteristics of Sr-HA under illumination, we computed the energy-loss spectra by converting the computed complex dielectric functions [49,50,51]. 

Accordingly, the energy loss function depicted in Figure 2b shows that doped apatites have a strong plasma frequency peak at approximately 18 eV. However, weak energy loss in the range of 355–415 nm (~3–3.5 eV) specifies that these fillers can provide an increased curing depth when they are used in a UV-LED cured dental composite.

Moreover, powders obtained via the IL-assisted approach show a relatively higher crystallinity compared to the other synthesis method. Materials with higher crystallinity (fewer crystal defects) result in lower photon absorption in the UV-Vis region, which is an important parameter for light curable dental composites, leading to effective absorption of photo-initiators. The average crystallite size of the products was estimated using the Scherrer formula and applied to the main XRD peaks [52,53,54], which reveals that the sample obtained via the ILH route (about 35–42 nm) has a lower average crystallite size compared to the sample prepared using the CH method (about 68–75 nm), which is caused via the role of IL in controlling the growth process. 

**Figure 3:**
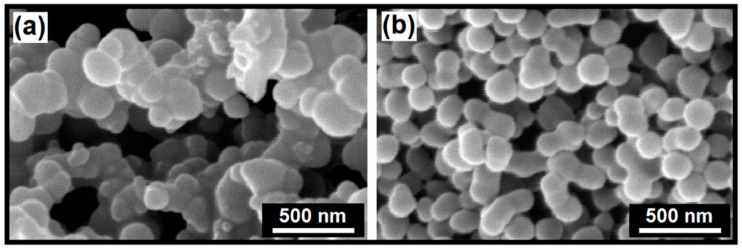
SEM images of the as-prepared products via hydrothermal method without (**a**) and with ionic liquids (ILs) (**b**).

**Figure 4:**
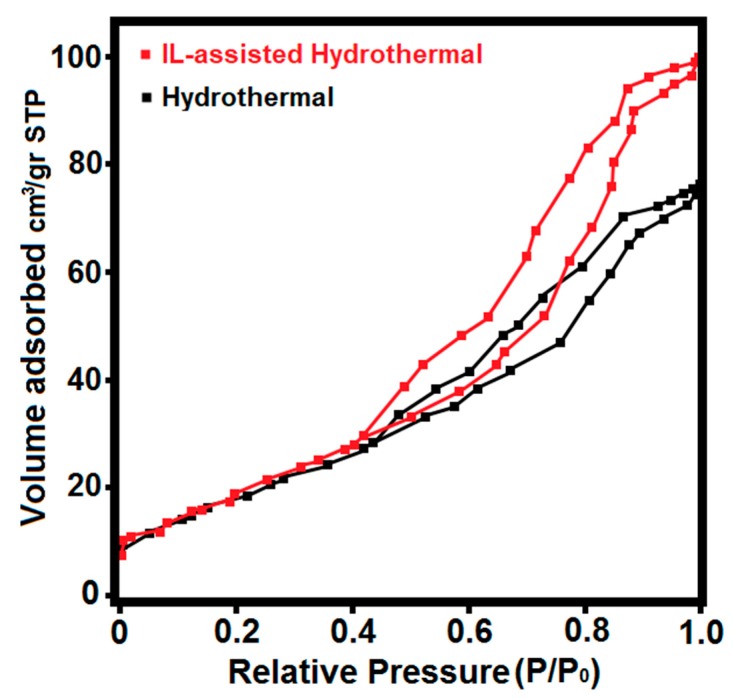
Nitrogen adsorption-desorption isotherm of the obtained products.

The morphology and microstructure of the as-synthesized products were characterized using SEM (as shown in Figure 3). The IL-derived product consists of uniform spherically shaped particles with sizes ranging from 100 to 200 nm. The metal atom coordination and surrounding of the particles with IL molecules as chelating and surface-passivating ligands cause the homogenous metal distribution and kinetic control of the particle growth. This leads to homogenous primary micro-particles and a narrow size distribution, without significant agglomeration after the calcination process. This in turn results in a high surface area (about 51.3 m^2^·g^−1^), which is consistent with the Brunauer–Emmett–Teller (BET) analysis with a type IV adsorption classification (presented in Figure 4). The significant surface area (about 50 m^2^/g) closely corresponds to the estimated small crystallite size of 30–40 nm, as determined from XRD. This is easily calculated from the relation between non-porous particle size (assumed spherical) and surface area, as follows: A (in m^2^/g) = 6000/(ρD), with ρ as the material density in g/cm^3^ and D as the particle diameter in nm. This immediately leads to the conclusion that the particles, as observed in SEM consist, of loose aggregates of a small number of non-porous crystallites.

In contrast, the sample prepared without IL shows irregular morphology with higher particle size (100–350 nm, as seen in Figure 3a) and massive agglomeration, leading to a lower surface area (about 37.1 m^2^·g^−1^). 

**Figure 5:**
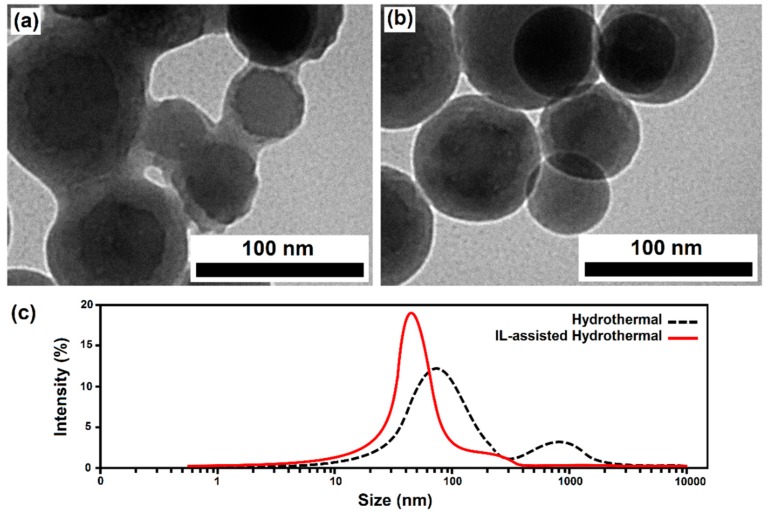
TEM images ((**a**) without ILs and (**b**) with ILs) and particle size distribution (**c**) of the as-prepared products via the hydrothermal method, without and with ILs.

Based on the above observations, it can be concluded that the addition of [BMIM]Br plays a key role in the formation of the smaller and regular spherical nanoparticles of HA powders with mesoporous structures. Accordingly, the main cations [BMIM]**^+^** can serve as capping agents and adsorb on different faces of crystals to reduce the surface energy of the polar planes, resulting in low-dimensional particles with a well-defined morphology. Moreover, the [BMIM]Br mainly acts as a crystal growth modifier, hereby controlling the ion diffusivity of the solution and the growth rate on different crystal surface planes, leading to the isotropic growth of nanoparticles with a uniform spherical shape (Figure 5b). Furthermore, the reaction was found to occur rather fast in a solvent with [BMIM]Br and the driving force would be pretty high due to the low interface tensions of the ionic liquid, thus resulting in higher nucleation rates and the formation of smaller nanoparticles [25,26,30]. The relatively larger surface area and the mesoporous architecture of the prepared sample via the ILH method can provide more active sites for adsorption and interaction of surrounding molecules, reinforcing obtained dental composites more effectively. 

According to the DLS measurements (Figure 5c), the average hydrodynamic particle size for IL-assisted hydrothermal (ILH) and conventional HT-derived samples was estimated at about 47 and 89 nm, respectively. It clearly shows a narrow size distribution for the IL-assisted method in comparison with the other preparation method. Moreover, the average hydrodynamic size of the nanoparticles measured by DLS is consistent with the TEM results. Bimodal size distributions were observed for the conventional hydrothermal-prepared sample, whereby the higher particle-size peak corresponds to clusters and the aggregation of smaller particles. Again, the presence of IL during the synthesis is important to achieve well dispersed nanoparticles and allows for the kinetically controlled growth of crystal faces of nanocrystals. 

**Figure 6:**
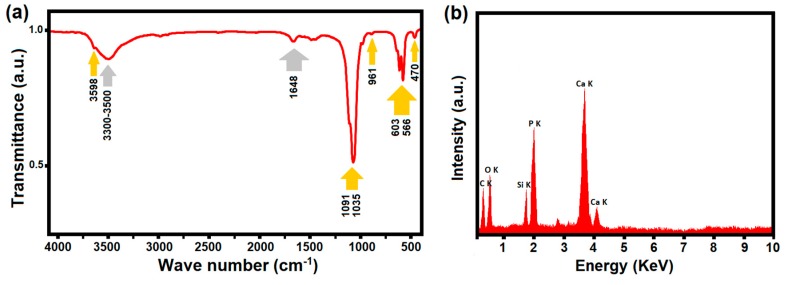
FTIR spectrum (**a**) and EDS analysis (**b**) of the as-prepared Sr-doped HA via the IL-assisted hydrothermal approach.

The FTIR spectrum (Figure 6a) showed the presence of OH, HPO_4_^2−^, and PO_4_^3−^ absorption bands, confirming the formation of HA obtained via the ILH method. Prominent peaks in the measured range of 400–4000 cm^−1^ were assigned to the bending vibrations mode (470, 566, and 603 cm^−1^), the non-degenerated symmetric stretching mode (961 cm^−1^), and the triply degenerated stretching mode (1035–1091 cm^−1^) of the P-O bonds. The peaks at 632 and 3598 cm^−1^ correspond to the stretching vibration of OH^−^ ions in the HA lattice, while the weak broad bands at 3300–3500 cm^−1^ and 1648 cm^−1^ are attributed to the adsorbed water [55,56,57,58]. 

Moreover, EDS was performed and the results confirm the presence of all elements in the ILH-obtained sample (Figure 6b)and the obtained Ca/P ratio was measured as 1.698, relatively close to the stoichiometric Ca/P ratio expected for a pure HA phase (1.67).

**Figure 7:**
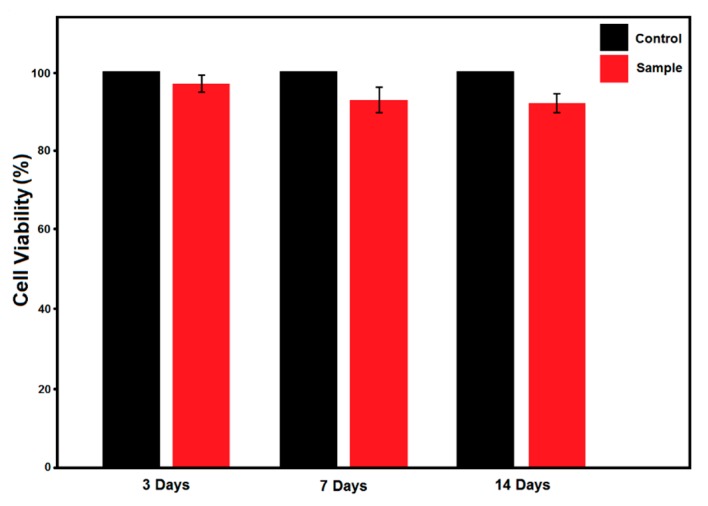
Cell viability of the sample prepared via IL-assisted hydrothermal method, for different periods of time, at 37°C, as measured by MTT assay.

Finally, the cytotoxicity of the ILH-prepared HA nanoparticles was also conducted using MTT assay and the results are presented in Figure 7. There are no significant differences in the proliferation rate of the cells in the presence of ILH derived HA powders compared to that of the control sample and the cellular viabilities were estimated greater than 91%, even after 14 days. The cytotoxicity test indicates that, without further surface modification, the as-synthesized Sr-doped HA nanoparticles showing acceptable biocompatibility as well as a relatively high surface area, therefore, have a good potential for use in UV-LED curable dental restorative composites.

## 4. Conclusions

In this work, Sr-doped HA nanoparticles was synthesized via a modified hydrothermal route with the assistance of [BMIM]Br ionic liquid and the resultant products were comprehensively characterized. The computational study shows that Sr-doped HA possessed weak energy loss in the range of 355–415 nm (~3–3.5 eV), which can provide an increased curing depth when it is used in a UV-LED cured dental composite. According to experimental results, the as-synthesized product indicated a significant surface area (about 50 m^2^/g) and an acceptable biocompatibility (greater than 91%). Therefore, the as-prepared Sr-doped HA nanoparticles present good potential for using in UV-LED curable dental restorative composites.

## Figures and Tables

**Table 1:** Experimental and computational calculated unit cell parameters of the as-prepared products.

	Cell Parameters	a = b (Å)	c (Å)
Calculated Data			
**Sr-doped HA (IL-assisted HT)**		9.5704	6.9980
**Sr-doped HA (Conventional HT)**		9.5812	7.0502
**Pure HA (IL-assisted HT)**		9.402	6.850
**Sr-doped HA (Computational method)**		9.6590	7.1521
**Pure HA (Computational method)**		9.6893	6.9762
